# Evaluating the Efficacy of Knowledge-Transfer Interventions on Animal Health Knowledge of Rural Working Equid Owners in Central Ethiopia: A Cluster-Randomized Controlled Trial

**DOI:** 10.3389/fvets.2018.00282

**Published:** 2018-11-20

**Authors:** Andrew P. Stringer, Rob M. Christley, Catriona E. Bell, Feseha Gebreab, Gebre Tefera, Karen Reed, Andrew Trawford, Gina L. Pinchbeck

**Affiliations:** ^1^Institute of Infection and Global Health, University of Liverpool, Liverpool, United Kingdom; ^2^Royal (Dick) School of Veterinary Studies, University of Edinburgh, Roslin, United Kingdom; ^3^College of Veterinary Medicine and Agriculture, Addis Ababa University, Debre Zeit, Ethiopia; ^4^Society For The Protection Of Animals Abroad (SPANA), London, United Kingdom; ^5^The Donkey Sanctuary, Sidmouth, United Kingdom

**Keywords:** randomized controlled trial, intervention, education, equid, Ethiopia, knowledge transfer, animal health

## Abstract

The objectives of this study were to evaluate the efficacy of several knowledge-transfer interventions about donkey health, utilizing a cluster-randomized controlled trial (c-RCT), on the long-term knowledge change (~6 months post intervention) of Ethiopian rural working equid owners. Knowledge transfer interventions included: an audio programme, a village meeting and a diagrammatic hand-out, which were also compared to a control group, which received no intervention. All interventions addressed identical learning objectives. Thirty-two villages were randomly selected and interventions randomly assigned to blocks of eight villages. All participants in a village received the same intervention, and knowledge levels were assessed by questionnaire administration both pre and post intervention. Data analysis included multilevel linear and logistic regression models (allowing for clustering of individuals within villages) to evaluate the change in knowledge between the different knowledge-transfer interventions, and to look at other factors associated with change in knowledge. A total of 516 randomly selected participants completed pre-intervention questionnaires, 476 undertook a post-dissemination questionnaire ~6 months later, a follow-up response rate of 92%. All interventions significantly improved the overall knowledge score on the post intervention questionnaire compared to the control group, with the diagrammatic hand-out [coefficient (coef) 10.0, S.E. = 0.5] and the village meeting (coef 8.5, S.E = 0.5) having a significantly greater impact than the audio programme (coef 4.0, S.E = 0.5). There were differences in learning across interventions, learning objectives, age and education levels of the participants. Participants with higher levels of formal education had greater knowledge change but this varied across interventions. In conclusion, knowledge of donkey health was substantially increased by a diagrammatic hand-out and the impact of this simple, low-cost intervention should be further evaluated in other communities in low-income countries. This study should assist in the design and development of effective knowledge-transfer materials for adult learning for rural villagers in low-income countries.

## Introduction

Working equids are increasing in numbers in many low-income countries, and their importance is being emphasized in response to increasing human populations, global economic issues, and changing environments (1). There are estimated to be 2.2 million horses, 0.41 million mules, and 8.4 million donkeys working in Ethiopia (2). The health, welfare and productivity of working horses, mules and donkeys in Ethiopia are affected by prevalent parasitic and infectious diseases, and problems associated with inadequate management practices (3–6).

There are numerous approaches to address the health and welfare impacts of wounds in working donkeys, one approach, is through the education of owners and communities. Stringer et al. (7) described the short-term knowledge change (~2 weeks after intervention) associated with three knowledge-transfer interventions on equid owners using a cluster-randomized controlled trial. However, few randomized controlled trials have evaluated longer term knowledge change of animal owners and it is important to understand whether knowledge on a specific subject decreases over time, as learning may decay unless reinforced (8). Grace et al. (8) evaluated the knowledge of cattle owners in Mali ~5 months after an educational intervention and demonstrated that their knowledge on a specific subject (cattle trypanosomosis) was reduced at 5 months when compared to the 2 week post intervention assessment. To reduce this knowledge fade at longer time intervals post intervention, it is recommended that information for owners be made readily and continually available to farmers (8).

The objectives of this study were to assess the efficacy of three knowledge transfer interventions (an audio programme, a village meeting and a diagrammatic hand-out) on the long-term (~6 months) knowledge change of participants and to assess if learning was different across different types of questions and learning objectives.

## Materials and methods

The content of the knowledge-transfer interventions and the design of the cluster-randomized controlled trial (c-RCT) has been described in detail in Stringer et al. (7). The study developed ten learning objectives (Table 1) based around key issues identified during an initial participatory situation analysis phase of the study (5). These issues were associated with causes, sites, treatment, prevention and relevance of donkey wounds and their management. The learning objectives in this study provided a defined educational framework around which all three of the different knowledge-transfer interventions were designed. These are available on request and included an audio programme (A), a village meeting facilitated by one trained animal health worker (VM), and a diagrammatic hand-out (HO). The results of other relevant published studies, including a participatory situation analysis undertaken at the beginning of this study (5), alongside future sustainability, economic and logistical considerations informed the selection of intervention formats. The target population in this study was expected to have both low levels of formal education and literacy (in both regional languages: Amharic and Afan Oromo), and this was accounted for in the design and development phase of the knowledge-transfer interventions (7).

**Table 1:** Learning objective (and the corresponding questionnaire number and question topic) used to provide a framework for developing three different knowledge-transfer interventions for rural working equid owners in Ethiopia relating to wounds and wound management.

	**Learning objective**	**Questionnaire number**	**Question topic**	**Maximum question score**
1	Be able to list four causes of manmade wounds.	2	C/S	4
2	Identify four common sites/areas affected by manmade wounds.	1	C/S	4
3	Be aware of good and bad topical treatments for wounds.	5, 6	T	1 + 3
4	Describe how to prepare an appropriate salt solution for cleaning wounds.	7	T	1
5	Be able to list three steps involved in cleaning wounds appropriately.	4, 8	T	2 + 1
6	Recognize two signs of an early harness wound.	3	C/S	2
7	Select appropriate material as a base layer for the harness.	9	P	3
8	Describe three important features of the padding on the harness.	10	P	3
9	Describe an important feature of harness base layer care.	11	P	1
10	Recognize three disadvantages of your donkey having wounds.	12	R	3

*C, Causes/Sites; T, Treatment; P, Prevention; R, Relevance*.

**Figure 1:**
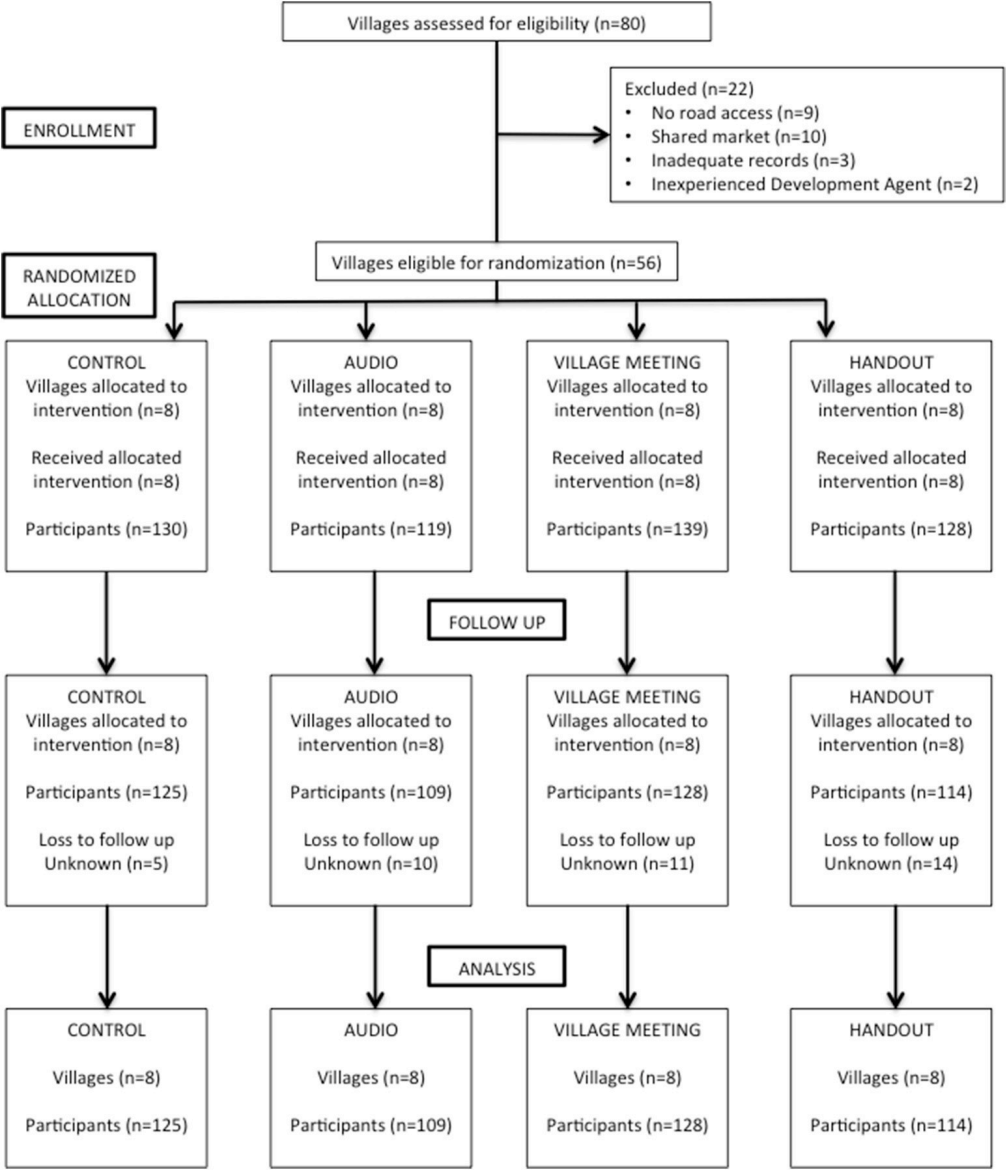
c-RCT flow diagram indicating number of participants and villages at each stage of the trial.

The effects of the three knowledge-transfer interventions on change in knowledge of rural working equid owners were compared with a control group (that received no knowledge-transfer intervention) using a c-RCT (Figure 1). The results from short-term follow-up at 2 weeks have been published previously (7), whilst this study evaluates results from the long-term follow-up. The c-RCT was carried out in the Oromia regional zone of Ethiopia where one zone (Arsi) was selected based upon: a lack of previous exposure to an equine veterinary NGO, a known high density of donkey users and logistical considerations. Four administrative departments (Sire, Hitosa, Tiyo, Degeluna Tijo) were convenience sampled based upon: a lack of previous exposure to an equine veterinary NGO, a known high density of donkey users and logistical considerations (7).

A complete list of villages within each administrative department was obtained from the relevant administrative departments agricultural office and 32 villages (kebeles) including 516 working equid owners were then randomly selected. Each village was assigned randomly to one of the three intervention groups or to a fourth control group, to give eight villages per group, and the same intervention was assigned to all participants within each village. Villages were excluded if they lacked any road access; the development agent (DA) was either new to that village or inexperienced; if villager records were inadequate; or if selected villages shared a major market, as this could potentially lead to contamination of the study design if participants shared information with each other at the market. Development agents were recruited to facilitate liaison with selected villages and to aid in the recruitment of participants. Lists of all village inhabitants were obtained from village agricultural offices or municipality offices, and individuals within each village were randomly selected using random numbers generated in Microsoft Excel 2007 (Microsoft Cooperation, USA). Randomly selected participants that did not meet the inclusion criteria as determined by the list information and development agent knowledge of household were excluded. Criteria for inclusion in the c-RCT included participants who were male, used or owned a donkey, >18 years of age, and had the ability to attend the study visits. All participants were free to refuse participation or to request to leave the trial at any point. Formal consent was assumed by continued participation in the trial following an introduction to the trial. Recruited participants were assigned an ID card with a unique identification (ID) number, and also received a nominal monetary incentive for participation in each study visit. Dates and times for pre-intervention visits and the follow up visits were determined at the outset of the study, and DA's were responsible for informing participants (7). One of the inclusion criteria for this study was gender, with only males being selected for participation. This decision was based on information gathered during the pilot phase that identified an existing male dominated hierarchy within Ethiopian households with the majority of decisions regarding use and healthcare of owned donkeys being made by males.

Identical questionnaires (Supplementary Informations 1, 2) were administered to all participants both pre- and post-dissemination to assess changes in knowledge levels. A cluster sample size calculator was used to perform sample size estimates for a clustered study design (9). The variance at village level was estimated from previous studies in developing countries (10), resulting in a design effect of 2.3 and an intra-cluster correlation coefficient of 0.14. A total of 15 owners in each of eight villages per type of intervention (total 480 participants) were required to detect a 30% change in knowledge (e.g., an increase from a baseline of 20 to 50%) with 95% confidence and 80% power. Thirty two villages with more than 25 owners per village were therefore selected to allow for potential non-response and loss to follow up. A blocked design was used such that, within each set of eight randomly selected villages, each knowledge-transfer intervention and control was assigned randomly to two villages. This design was adopted to mitigate runs of one type of intervention being selected by chance, as we hypothesized that seasonal job activities of farmers, may affect the response rates. Long term follow up questionnaires were administered 138–196 days post intervention (median 141 days).

### Data collection

Baseline data were gathered at the pre-intervention visit including age, formal education level, radio access, levels of literacy (in Afan Oromo and Amharic languages), number of donkeys owned, length of donkey ownership, other animals owned, housing of donkey, exposure to equine veterinary non-governmental organizations (NGOs) and position in household. Participants' baseline knowledge was measured using 12 concise questions regarding donkey wounds and wound management. These questions corresponded directly to the ten defined learning objectives (Table 1), and identical questions were used in both pre-intervention and follow-up questionnaires. The participants were scored using two methods both of which were used in analysis. Firstly, an overall score producing a continuous outcome was calculated. The 12 questions required participants to volunteer between one and four correct responses per question. For example, one question asked participants: “What are the causes of manmade wounds of donkeys.” For this question, there were four possible causes, a mark was gained for each of the four correct causes (a maximum of four marks for this question). Participants could therefore score between zero and four on this question. The continuous outcome was out of 28 (the maximum score for all the individual parts of each question). Each question was also scored as correct or incorrect (binary outcome) depending on whether the participant correctly answered all the individual parts or not, respectively. For example, the participant would have had to correctly volunteer all four causes of manmade wounds to get that question correct. The dataset for this manuscript is not publicly available because an institutional ethical approval for open access data was not required at the time when this study was designed and conducted. Requests to access the dataset should be directed to Dr. Andrew Stringer (apstringer@ncsu.edu). Data will be made available on successful completion of an institutional ethics application.

### Data analysis—multilevel linear regression analysis

Data were analyzed using SPSS v19 (SPSS Inc, Chicago, Illinois, USA) and MLwiN v2.25 (Centre for Multilevel Modeling, Bristol, UK). Data analysis included comparison of baseline data between intervention groups to check for adequate randomization using Chi-squared tests for categorical data and Kruskal-Wallis or Mann-Whitney tests for continuous data (7). The outcome measure used was a continuous variable reflecting the change in score between pre- and post-intervention questionnaires (out of a maximum of 28). The change in score of individual respondents was compared between the different knowledge-transfer interventions using multilevel linear regression models to allow for clustering of individuals within villages. Analysis was carried out on a per-protocol basis due to no data being available on the outcome of those participants lost at follow up. The effect of all covariates that varied at baseline was also considered.

All variables that showed some association with the outcome on univariable analysis (*p* < 0.25) were considered during the building of the final multivariable models. Continuous variables (age and pre-intervention score) were centered by subtraction of the sample mean from all observations and checked for linearity before entry into the final model by use of a generalized additive model (GAM) (11). A backward-stepwise process was used, with covariates remaining in the model if they were statistically significant (*p* < 0.05), or if they altered the effect of other covariates by >25% (Table 2) (7). Random slopes, interactions terms and model diagnostics (including evaluation of residual plots) were all assessed as previously described.

**Table 2:** Multilevel linear regression models showing the impact of different interventions on a change in knowledge score between questionnaires in 476 participants in a c –RCT in Oromia region, Ethiopia at long-term follow-up (138–196 days post intervention).

	**Model 1**		**Model 2**	
	**Coefficient (S.E)**	***P*-value**	**Coefficient (S.E)**	***P*-value**
**INTERVENTION**				
Control (intercept)	0.8		0.8	
Audio	4.0 (0.5)	<0.001	4.0 (0.5)	<0.001
Handout	10.0 (0.5)	<0.001	10.0 (0.5)	<0.001
Village meeting	8.6 (0.5)	<0.001	8.5 (0.5)	<0.001
Age (years)^a^			−0.04 (0.02)	<0.001
Pre-intervention score^a^			−0.5 (0.06)	<0.001
INTERACTION: INTERVENTION^*^AGE^a^				
Control^*^Age^a^			Ref.	
Audio^*^Age^a^			−0.006 (0.03)	0.8
Handout^*^Age^a^			−0.07(0.03)	0.008
Village Meeting^*^Age^a^			−0.008 (0.03)	0.8
Village variance	0.4 (0.2)		0.4 (0.3)	
Individual variance	9.0 (0.6)		8.7 (0.6)	

a Indicates variables were centered. The control coefficient (intercept) represents the change in score for controls of average age and with average pre-intervention score. Ref., Reference category.

*Model 1: This model only considers the interventions. Model 2: This model considers the interventions and those covariates shown to have a significant effect on the outcome*.

### Data analysis—multilevel logistic regression analysis

The outcome measure used was a binary variable reflecting whether knowledge improved for each of the 12 individual questions. Hence, where participants answered incorrectly at the pre-intervention questionnaire and correctly at the post-intervention questionnaire they were deemed to have improved knowledge and were coded as one. All other combinations were coded zero (Supplementary Information 3 and Table 3). For the majority of questions, few participants got the answer correct first time, so the number of participants going from correct to incorrect, or correct to correct was low (Supplementary Information 3). Associations between the dependent variable (knowledge improvement of individual participants) and the independent variables including the intervention type, the question topic (causes and signs, treatment, prevention, relevance) the learning objective, education levels, age and other demographic variables were compared using three-level logistic regression models to allow for clustering of questions within individuals, and individuals within villages. Where variables were highly correlated, a decision was made to include only one of the variables into each separate model, or to include the most biologically meaningful variable. All variables that showed some association with the outcome on univariable analysis (*p* < 0.25) were considered during the building of the final multivariable models. A backward-stepwise process was used, with covariates remaining in the model if they were statistically significant (*p* < 0.05), or if they altered the effect of other covariates by >25%. Models were fit using penalized quasi-likelihood with 2nd order Taylor series expansion. The significance of random slopes and interaction terms were tested between all fixed effect variables. In order to fit models, it was necessary to remove certain variables due to small sample sizes. For example, for certain learning objectives (learning objectives one, six, eight, and ten) only a small number of participants had improved their knowledge at long term follow up and this made it problematic to fit interaction terms. As a result, only learning objectives where >10% of participants had improved their knowledge were included in the final model. Plots of predicted probabilities (predicted probability of getting a specific question correct after an intervention) were used to demonstrate significant interaction effects between independent variables in the final model.

**Table 3:** Percentage of participants (*n* = 476) who improved on specific learning objectives across interventions groups between pre-intervention and long-term follow-up.

	**Learning objectives**	**Improved control *n* (%)**	**Improved audio *n* (%)**	**Improved village meeting *n* (%)**	**Improved handout *n* (%)**	**Improved all *n* (%)**
1	Be able to list four causes of manmade wounds.	0 (0.0)	3 (2.8)	16 (12.5)	18 (15.8)	37 (7.8)
2*	Identify four common sites/areas affected by manmade wounds.	7 (5.6)	42 (38.5)	99 (77.3)	90 (78.9)	238 (50.0)
3*	Be aware of good and bad topical treatments for wounds.	19 (7.6)	89 (40.8)	196 (76.6)	184 (80.7)	488 (51.3)
4*	Describe how to prepare an appropriate salt solution for cleaning wounds.	0 (0.0)	8 (7.3)	51 (39.8)	69 (60.5)	128 (26.9)
5*	Be able to list three steps involved in cleaning wounds appropriately.	10 (4.0)	42 (19.3)	105 (41.0)	97 (42.5)	254 (26.7)
6	Recognize two signs of an early harness wound.	0 (0.0)	0 (0.0)	1 (0.8)	4 (3.5)	5 (1.1)
7*	Select appropriate material as a base layer for the harness.	0 (0.0)	0 (0.0)	11 (8.6)	55 (48.2)	66 (15.5)
8	Describe three important features of the padding on the harness.	0 (0.0)	1 (0.9)	11 (8.6)	6 (5.3)	18 (3.8)
9*	Describe an important feature of harness base layer care.	13 (10.4)	19 (17.4)	43 (33.6)	50 (43.9)	125 (26.3)
10	Recognize three disadvantages of your donkey having wounds.	0 (0.0)	0 (0.0)	2 (2.3)	11 (9.6)	14 (2.9)

* *, learning objective (with >10% of participants improving) included in multilevel regression analysis*.

## Results

### Descriptive results

The pre-intervention questionnaire was completed by a total of 516 participants from 32 villages (Figure 1). There was a loss of 40 participants at the long-term follow-up phase, thus 476 participants completed the post-intervention questionnaire, an overall response rate of 92% (Figure 1). No significant difference was identified in the proportion of participants lost across intervention groups. Baseline information regarding participants revealed low formal education levels, with the majority (72.4%) of participants having only formally attended to primary school level (7). There were low levels of literacy (41.5% of participants were not literate in either language), with a greater proportion of participants unable to read Afan Oromo (78.5%) than Amharic (21.5%). The majority of the participants had access to a radio (80.0%).

### Baseline comparison of data

Analysis of baseline data to check the randomization process showed that a number of variables (including age, number of donkeys owned and ownership of horses, sheep, goats, and dogs) were significantly different between intervention groups (*P* < 0.05), and pre-intervention scores approached significance (*p* = 0.08) (Supplementary Information 4).

### Change in score between pre- and post-intervention questionnaires

Change in score was approximately normally distributed and ranged from −5 (i.e., some participants had a lower score at follow up) to 19. The median scores and interquartile range (IQR) at the pre-intervention stage for each intervention were: Control (6.75, IQR = 5.00−8.00), Audio (7.00, IQR = 5.00−8.00), Handout (6.00, IQR = 5.00−7.00), and Village Meeting (6.00, IQR = 4.00−8.00). The median scores and interquartile range (IQR) at the post intervention stage were: Control (7.00, IQR = 6.00−8.50), Audio (11.00, IQR = 9.00−13.00), Handout (17.50, IQR = 13.86−20.50), and Village Meeting (15.50, IQR = 12.63−18.38). All intervention types were considered in the final model (Model 2 in Table 2), and those covariates which had a significant effect on the outcome (age and pre-intervention score). All interventions resulted in a significant improvement in the overall change in score between pre- and post-intervention questionnaires compared to the control (Table 2), with the hand-out and village meeting interventions demonstrating a significantly greater impact on knowledge change than the audio programme (*p* < 0.001). There was also a significantly greater increase in knowledge with the hand-out compared to the village meeting (*p* = 0.02).

**Figure 2:**
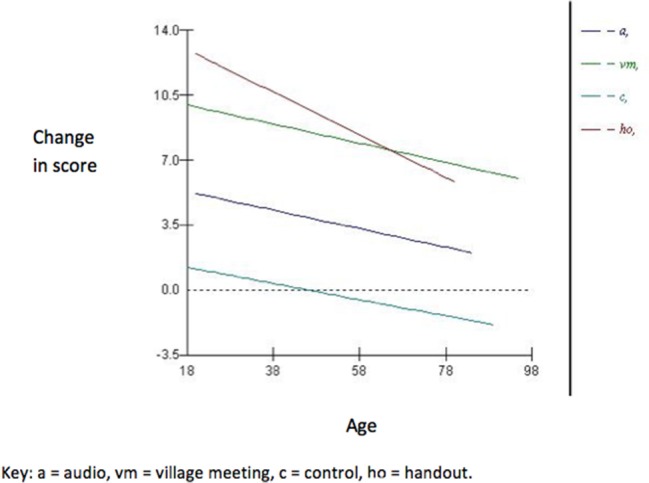
Plot showing the effect of the significant interaction between age and interventions in the multilevel linear regression model (Model 2).

The increase in knowledge was lower amongst older participants, with a significant interaction identified between age and the effect of the intervention on knowledge change (Figure 2), showing that the effect of a decreasing change in score with age was more pronounced in the hand-out group. The variance at the village level was small and accounted for only 4.3% of the total variation. No significant random slope effects were identified, suggesting that there were no differences in the effect of a single type of intervention across different villages. Normal probability plots of both the village and individual level residuals demonstrated that the assumption of normality was reasonable. Village level residual plots identified one village was significantly different from the overall mean. This village had received a handout intervention. Plots of leverage and influence values also highlighted that this village had moderately high leverage and the highest influence value. Inclusion of this village as a pacifier variable, as described by Rasbash et al. (12), to fit an intercept separately from those of the other villages did reduce the overall deviance and the parameter estimate for this village was significant (coefficient −2.7, S.E 0.9), demonstrating that the change in score was decreased in this village. However, estimates for the overall effect of the interventions changed very little (7).

### Knowledge improvement for individual questions

At long-term follow up, an improvement in knowledge on eight of the 12 questions was demonstrated by >10% of participants, with the other four questions having < 10% of participants improving knowledge (Supplementary Information 3). Six of the ten learning objectives had >10% of participants improving, and are highlighted in Table 3. There were variations across each question and each learning objective in the percentage of participants that improved depending on which intervention they received (Supplementary Information 3 and Table 3). The largest overall improvement was seen in learning objective 3 (Be aware of good and bad treatments for wounds). This learning objective corresponded to two questions in the questionnaire (questions 5 and 6). Overall, 51.3% of participants improved their knowledge on this learning objective, with 80.7, 76.6, and 40.8% of participants who received a hand-out, a village meeting or audio programme improving, respectively. Two of the learning objectives (learning objectives 4 and 7) revealed much larger improvements in knowledge in participants who received a hand-out, compared to participants who received an audio programme. For learning objective 6, participants were required to answer that both pain and hair loss were two of the early signs associated with a harness wound. Following interventions, many participants (*n* = 276) were able to answer hair loss, however only a few (*n* = 5) were able to answer pain. Learning objective 8 (a prevention question), was answered correctly by < 10% of participants. This learning objective required participants to correctly describe three important features of the padding on the harness. Again, many participants could name one or two of these features, but were unable to name all three features. The “relevance” question topic was represented by only one question (and learning objective 10). Participants were required to recall three disadvantages to their donkey having wounds. Again, very few individuals could recall all three correct responses. Overall, the most effective question topic in the three intervention groups was “treatment,” with 35.0% of participants improving in this question topic compared to “causes/signs” (19.6%), “prevention” (15.2%), and “relevance” (2.9%).

The explanatory variables identified by univariable analysis (*p* < 0.25) for consideration in the multilevel models were: intervention, age, radio access, Afan Oromo and Amharic literacy, formal education level, cattle/ox, sheep, mule, and dog ownership, whether an owner gave advice on donkey care, question number, question type, and learning objective. The final multivariable model showed that all interventions significantly improved the participants' ability to answer a question correctly at long-term follow-up, with the hand-out performing most effectively, followed by the village meeting and then the audio programme, when compared to the control (Table 4). Other significant variables in the final model included the learning objective, education level, and age. As per previous results, as age increased participants were less likely to have an improvement in knowledge. The final three-level logistic regression model revealed two significant interaction terms that suggested learning also varied learning objective and education level, with significant interaction terms in the model, with the effects of the interventions being different across different learning objectives and different education levels (Table 4).

**Table 4:** Multilevel, multivariable, binary regression models showing the factors associated with improving knowledge at long-term follow-up in 476 participants in a c –RCT in Oromia region, Ethiopia.

	**Odds ratio**	**Lower 95% CI**	**Upper 95% CI**	***P*-value**
Control	Ref			< 0.001
Audio	6.01	2.35	15.38	
Handout	26.50	10.63	66.05	
Village Meeting	23.97	9.43	60.94	
Age (years)	0.99	0.98	1.00	< 0.001
Education				
No Education	Ref			< 0.01
Adult Education	0.94	0.24	3.61	
Primary	0.52	0.21	1.29	
Junior	0.38	0.11	1.32	
Higher	1.04	0.39	2.79	
Learning Objective				
LO3	Ref			0.02
LO2	0.97	0.37	2.55	
LO4	0.09	0.01	0.87	
LO5	0.50	0.21	1.16	
LO7	0.09	0.01	0.87	
LO9	1.43	0.65	3.19	
Learning objective—intervention interaction				< 0.001
Audio.LO2	1.26	0.43	3.73	
Village Meeting.LO2	1.47	0.49	4.46	
Handout.LO2	1.23	0.39	3.87	
Audio.LO4	1.01	0.10	10.82	
Village Meeting.LO4	1.72	0.18	16.69	
Handout.LO4	3.28	0.33	32.40	
Audio.LO5	0.62	0.24	1.63	
Village Meeting.LO5	0.35	0.13	0.89	
Handout.LO5	0.24	0.09	0.64	
Audio.LO7	1.16	0.11	12.19	
Village Meeting.LO7	0.20	0.02	2.11	
Handout.LO7	1.69	0.17	16.68	
Audio.LO9	0.20	0.07	0.54	
Village Meeting.LO9	0.08	0.03	0.21	
Handout.LO9	0.09	0.03	0.24	
Education level—intervention interaction				0.02
Audio.adult education	1.99	0.44	8.98	
Village Meeting.adult education	2.28	0.52	9.93	
Handout.adult education	2.78	0.63	12.25	
Audio.primary education	1.93	0.65	5.76	
Village Meeting.primary education	3.74	1.34	10.39	
Handout.primary education	5.63	1.98	16.00	
Audio.junior education	4.30	1.04	17.76	
Village Meeting.junior education	4.51	1.11	18.36	
Handout.junior education	13.01	3.03	55.83	
Audio.higher education	1.29	0.39	4.27	
Village Meeting.higher education	1.98	0.58	6.75	
Handout.higher education	5.09	1.58	16.43	

*Key: LO2, Learning objective 2; LO3, Learning objective 3; LO4, Learning objective 4; LO5, Learning objective 5; LO7, Learning objective 7; LO9, Learning objective 9*.

**Figure 3:**
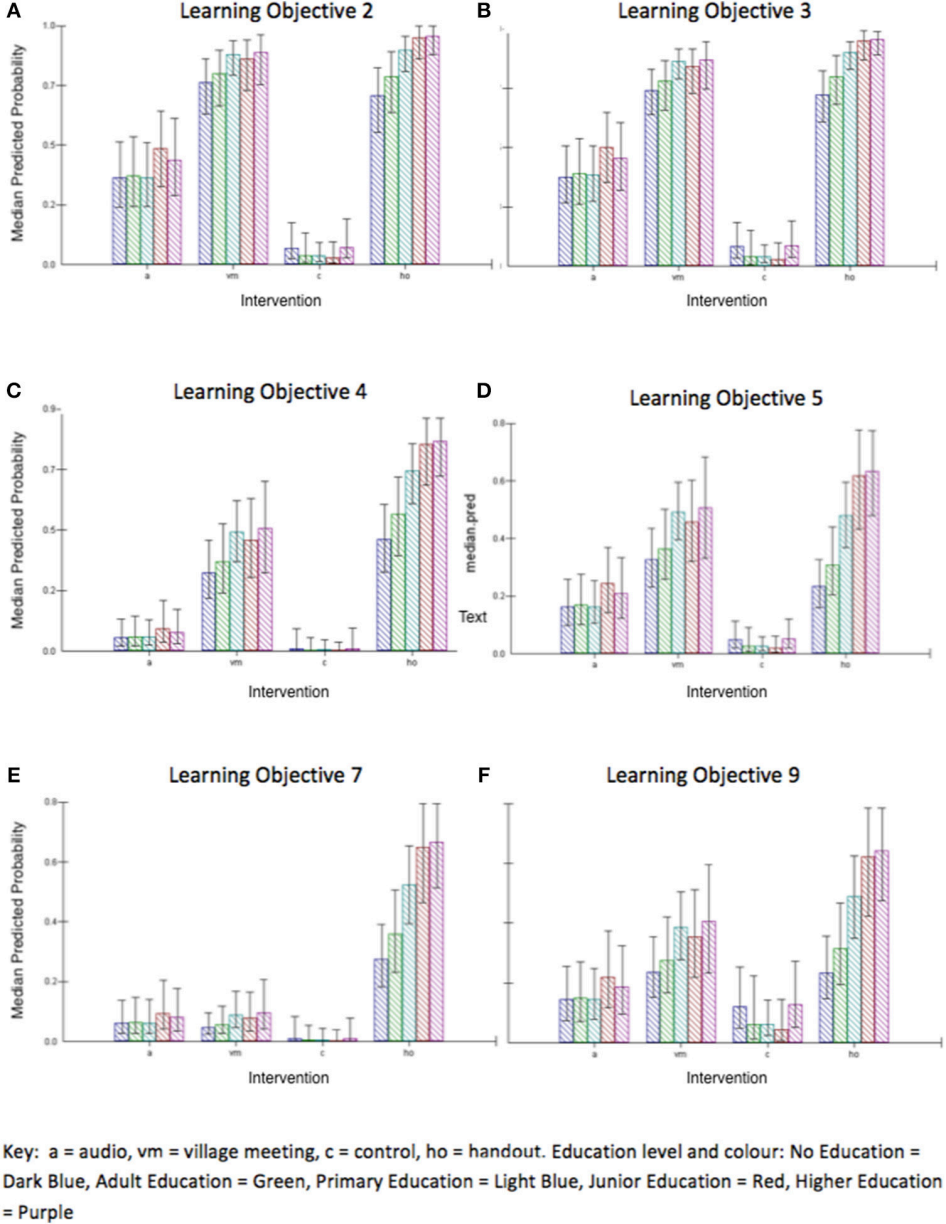
Predicted probabilities of getting a specific question correct for each intervention across different education levels and learning objectives.

**Figure 4:**
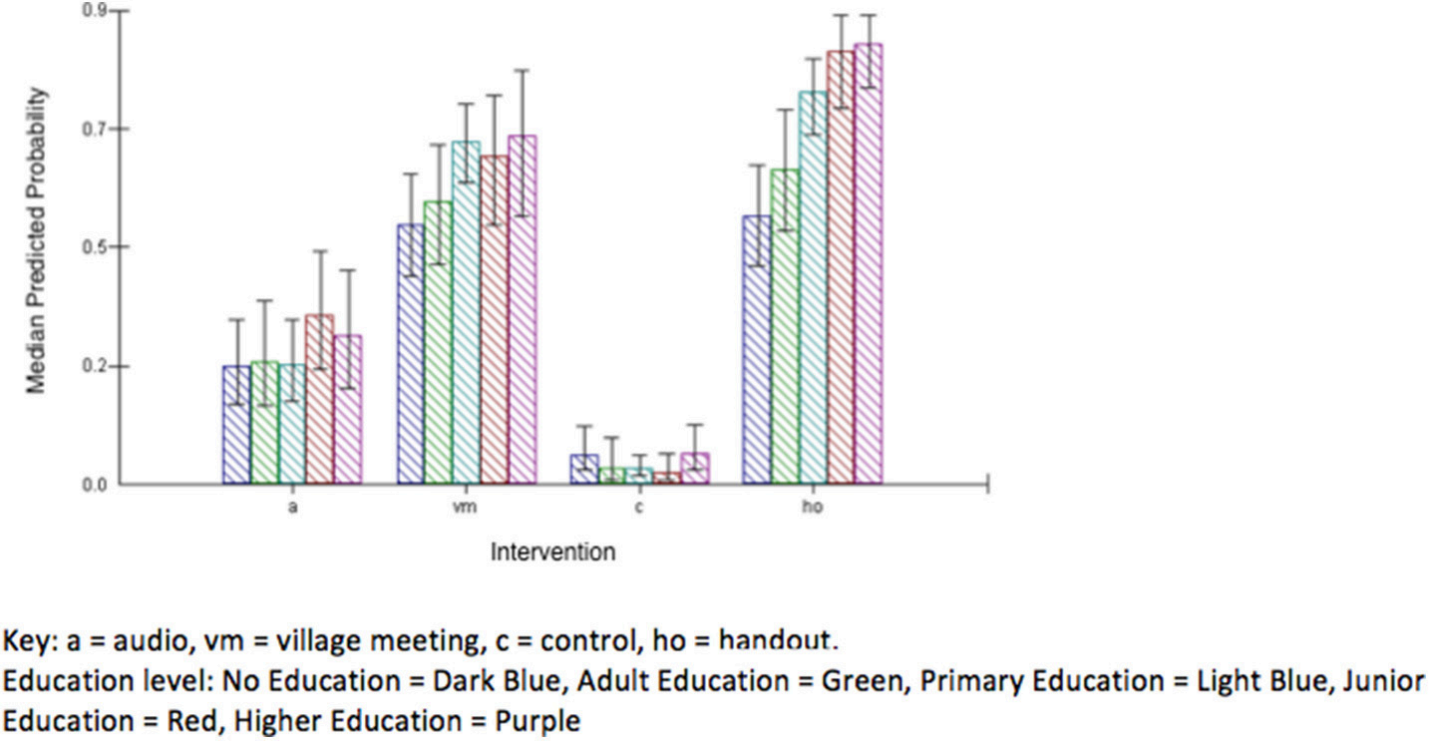
Predicted probability of a correct answer for each intervention and education level across average learning objective.

Plots of predicted probabilities (predicted probability of getting a specific question correct after an intervention) demonstrate this (Figures 3A–F). For example, with regards to learning objective 5 (Be able to list 3 steps involved in cleaning wounds appropriately), and learning objective 9 (Describe an important feature of harness base layer care) there is clearly an effect of education level in the hand-out intervention, but little or no effect of education in the audio or village meeting groups (Figures 3D,F). In learning objective 2 and 3, both village meeting and hand-out are similarly effective, with only slight effects of increasing effectiveness in participants with higher education levels (Figures 3A,B). With learning objective 4, the hand-out intervention is more effective than the village meeting, with a significantly greater effect at higher educational levels (Figure 3C). In learning objective 7 the hand-out is the only intervention that has any significant effect, again with a significantly greater effect at higher educational levels (Figure 3E). The predicted probability of getting a correct answer for each intervention and education level across average learning objective is presented in Figure 4. Interestingly, the mean age (57 years) of the lowest education group (no education) was 20 years older than the mean age (37 years) of the highest education group (higher education). The variance at the village level (0.014) was small compared to variance at the individual participant level (0.471) and accounted for only 2.9% of the total variation, demonstrating that the majority of the variance can be attributed to differences between individuals rather than between villages. Where possible, random slope effects were tested and were not significant, suggesting that there were no differences in the effect of a single type of intervention across different villages (7).

## Discussion

A limited number of studies have attempted to utilize randomized controlled trials to assess the impact of knowledge-transfer interventions on their target populations (7, 8, 10, 13). In this study, all interventions improved the knowledge of our target audience ~6 months (long-term) post-intervention. Overall, the hand-out was the most effective intervention, followed by the village meeting and then the audio programme. This is largely consistent with the previously published outcomes seen within the same study 2 weeks (short-term) after a follow up questionnaire (7). However, the change in knowledge in participants who received the village meeting or audio programme was smaller at the long-term follow-up, than at short-term follow-up. The hand-out, however, produced a marginally greater change in knowledge at the long-term follow-up than it did at the short-term follow-up. Given that the interventions were not repeated (village meeting and audio programme), some decrease in knowledge would be expected over a time and therefore could explain the reduction in knowledge at long-term. This is consistent with the study by Grace et al. (8) in which the change in farmer's knowledge decreased from that seen at the 2-week follow-up (31%), to 19% at the 5-month follow-up. The hand-out intervention was the only intervention that remained with the participants and therefore could have been referred to by participants prior to the follow-up, or at any time in between. This could have reinforced their knowledge on the subject matter, and therefore may explain the increase in knowledge at long-term follow-up from that seen at short term follow up.

The effect of age varied across interventions and was more pronounced in participants that had received the hand-out. The hand-out performed most effectively amongst younger participants, whilst in the older participants the village meeting was most effective. Again, this finding is consistent with the findings seen in the 2-week follow-up (7). Desjardins (14) suggested that older age groups have lower literacy proficiency than younger adults, most likely as a result of younger adults having received more extended formal education and more recently than older adults. Lower literacy proficiency could potentially result in a reduced ability for knowledge acquisition from knowledge-transfer interventions requiring literacy and/or visual literacy such as a hand-out. The use of defined learning objectives to design and develop the three specific knowledge-transfer interventions was crucial as they provided a defined educational framework around which all of the interventions were designed and ensured the content of each of the interventions was consistent, could be objectively evaluated and provided an overview of what the “learner should have achieved and what should be assessed” (15, 16). The choice of verb in a learning objective is key and defines what the learner should be able to do by the end of the training (rather than just a broad question topic).

This study identified variation in learning between question topic and learning objectives and furthermore, that this varied by intervention. It was hypothesized that the question topic would have an influence on how well participants learnt, and that the participants would have a greater knowledge change in the “treatment” question topic compared to the other question topics. The three learning objectives that related to “treatment” all revealed a significant change in knowledge in the intervention groups when compared to the control group. In contrast, at least one learning objective in each of the other three question topics (causes/sites, prevention, and relevance) was excluded from the model due to the small number (<10%) of participants improving at long-term follow-up on that learning objective. Learning objective 2, required owners to identify the four common sites of wounds, and this was answered well following the village meeting and hand-out interventions, with an overall improvement across all interventions of 50% of participants. The single visual aid that was used in both the hand-out and village meeting to teach this learning objective was designed to be clear, concise and easy to understand. By contrast, the other two learning objectives in the “causes/sites” question topic (learning objectives one and six) performed badly.

The target population in this study was expected to have both low levels of formal education and literacy (in both regional languages: Amharic and Afan Oromo), and the design and development phase of the knowledge-transfer interventions aimed to take account of this (7). This influenced the decision to produce a hand-out that was predominantly pictorial and diagrammatic in design. The participants in this study were found to have low levels of literacy, with a higher percentage of participants unable to read Afan Oromo than Amharic. However, the decision was taken to design the interventions in Afan Oromo, based on advice from local contacts, as this is currently the official language of the region, the language currently being taught in the region's schools and the language that the majority of our target population communicate in (despite being illiterate in this language). Children in this region currently attending school would be taught in Afan Oromo, and consequently would be literate in Afan Oromo during early primary education.

The success of the hand-out in this study highlights that the efforts made during the development and piloting phases resulted in an intervention that was, at least to some extent, appropriate and understandable to the visual literacy level of the study population. However, the effect of the hand-out did appear to vary with the age of the participant. The hand-out was more effective in younger participants than older participants even though efforts had been made to ensure the intervention was predominately image-based with limited text. Future knowledge-transfer programmes may need to be delivered using more than one medium, and may consider adopting village meeting formats for older and less formally educated individuals, whilst hand-outs could be utilized for younger and more formally educated individuals.

It is possible that the combination of an oral presentation with demonstrations and visual images (village meeting) may have accommodated all levels of literacy, visual literacy, and language issues. Immediately after the village meeting there was a question and answer session, this allowed participants an opportunity to clarify any areas of confusion or any missed messages in either of the two languages. The audio programme required no literacy ability, and was designed to simulate a possible future radio broadcast. Farr et al. (17) identified high levels of radio ownership amongst Ethiopian households, with regular radio listeners, which is consistent with this study demonstrating that 80% of participants had access to radio on a daily basis. Chapman et al. (18) found that radio formats that involved drama sketches performed by local actors were most popular amongst farmers listening to agricultural extension programmes, and that for maximum impact, the programme format should incorporate ways in which the intended target audience discuss problems in their own communities, and provide relevant information in a suitable context. This was demonstrated in our study by participants volunteering information about the storyline of the programme and the characters names. In this study, the audio programme was only played once to the participants, as such the authors did not explore the potential benefits to increased knowledge acquisition by repeat exposure to the intervention. The efficacy of this format was shown to be greatest amongst uneducated (no formal education) villagers in The Gambia (19). However, in this study there was no significant difference in the predicted probability of volunteering a correct answer for the audio intervention across education levels. Although the radio programme produced the smallest change in knowledge, when compared to the other two interventions it still significantly improved knowledge when compared with the control villages. The benefits of a successful audio intervention are its ability to “reach” thousands of individuals and households in a local language (many of whom may be illiterate) with relative ease of administration and low cost. These benefits may therefore outweigh the greater knowledge change efficacy seen in more labor-intensive interventions. The benefits may be enhanced through repeated exposure to the intervention (i.e., through repeated broadcast) and this should be further evaluated.

The questionnaire used for evaluating a change in knowledge utilized concise questions, many requiring only single word answers. However, the accuracy of information gathered during these questionnaire interviews must be considered carefully, especially as all information gathered went through a translation process. The reliability of information volunteered by participants was not validated and therefore may be imperfect or biased by participant reporting of perceived correct answers. However, due to the study design (c-RCT), we would expect this bias or measurement error to be randomized across all participants, in all intervention groups, and therefore to have minimal effect on the estimates of the effects of interventions.

The average improvement for a participant in the control group was only 0.8 marks. Hence, there was no evidence of the “Hawthorne-type Effect,” which occurs when there is a change in respondents (behavior) as a result of their involvement in the study, rather than due to the specific intervention (20). This study was designed to measure knowledge change within a target population, with all three interventions showing effective increases in knowledge when compared to the control intervention. Whilst changes in attitudes and behavior were not specifically measured in this study, the knowledge-transfer programme utilized in this study could be considered an initial step toward behavior change, with other components such as skills development, attitude development, and motivational support being required (21).

## Conclusion

Knowledge-transfer interventions developed for rural working equid owners (rural farmers) in this region of Ethiopia should consider the formal education level, and age of intended target audience as key issues, along with intervention type, and the educational learning objectives. This study showed that a hand-out, based primarily on visual images, and designed with the intended audience was demonstrated to be the most effective intervention, particularly in younger, and higher educated participants. The village meeting intervention, with direct contact between a specifically trained animal health worker and participants, in combination with a mixture of visual demonstrations, presentations and a question, and answer session was also effective. Ethiopia, with its large population of working equids and livestock is ideally placed to benefit from appropriate and effective animal health knowledge-transfer programmes. The results from this study may be beneficial to other populations of livestock owners, in other regions in Ethiopia, and across sub-Saharan Africa. However, it is possible that different considerations associated with learning across different communities may exist. Future studies should evaluate whether the impact of this simple, low-cost intervention can be replicated in other rural communities in low-income countries leading to measurable improvements in animal health.

## Ethics statement

The study was carried out under good ethical practice which included participants recruited on a volunteer basis. The study was non-invasive and participants were informed of the study objectives and that they were free to refuse participation or leave the trial at any point. Due to low levels of literacy, written informed consent was not obtained and consent was assumed by continued participation in the trial. All data were securely stored and identifying information removed for analysis. Animals were not used in the study. Given the nature of the study and the lack of formal ethical approval procedures at the Addis Ababa University at the time the study was designed and conducted, formal ethical approval was not obtained. However, the Faculty of Veterinary Science, Addis Ababa University, Debre Zeit, Ethiopia, were aware of and provided support to the study.

## Author contributions

AS, RC, CB, FG, GT, KR, AT, and GP conceived and planned the research. AS and GT conducted the research and data collection. AS, RC, CB, and GP contributed to the data analysis and interpretation of the results. AS took the lead in writing the manuscript. All authors provided critical feedback and helped shape the research, analysis, and manuscript.

### Conflict of interest statement

The authors declare that the research was conducted in the absence of any commercial or financial relationships that could be construed as a potential conflict of interest.
