# Supplementary material for: Evaluating the Efficacy of Knowledge-Transfer Interventions on Animal Health Knowledge of Rural Working Equid Owners in Central Ethiopia: A Cluster-Randomized Controlled Trial
Source: Front Vet Sci. 2018 Nov 20;5:282. doi: 10.3389/fvets.2018.00282 (PMC6256087; doi:10.3389/fvets.2018.00282)
Supplement: Supplementary file 1 [file Data_Sheet_1.PDF]

Working Equids Education Project

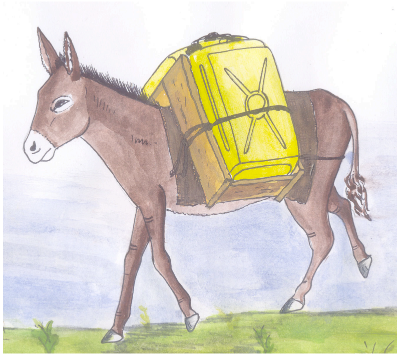

PRE INTERVENTION QUESTIONNAIRE

ID Number:	Name:
Date:	Gender:
DA Name:	Village (Kebele):

Method of education:

C	HO	VM	AO

Section 1: Background Information:

1. How old are you?

2. What is your responsibility in the family?

Husband		Son	
Wife		Daughter	
Other			

3. Who is responsible for the problems of donkeys?

Husband		Son	
Wife		Daughter	
Other			

Section 2: General Questions:

4. How many donkeys do you currently have?

5. Do you have any other animals?

Cow/Ox		Sheep	
Horse		Goat	
Mule		Dog	
		Poultry	

6. When did you begin using your own donkey?

7. Do you keep your donkey in "mooraa" during the night?

Yes / No

8. Have you taken your donkeys to whom come to your village to treat donkeys or to get education about donkeys?

Yes / No

If yes:

8a. Who were those? What did they do?

Section 3: Wounds:

9. On which body part of the donkey that you are observing manmade wounds most frequently? (*Picture aid*)

10. What are the causes of manmade wounds of donkeys?

11. Tell us two signs observed on donkeys before a wound is occurring?

Section 4: Wound treatment:

12. What care should be taken before you wash the wounds of donkeys?

13. Tell us the best way that you should use to cure the wounds of donkey?

14. Tell us the bad ways that should not be used to treat the wounds of a donkey?

15. To wash wound of donkeys, how much salt and how much water should be mixed?

16. How many times a day you should wash wound of donkeys?

Section 5: Prevention:

17. What type of “baselayer” you have to use for your donkeys?

18. To make the harness good and protect the donkey from the load tell us 3 things you should observe?

19. For the “baselayer” of the donkeys what care should be taken?

20. What are the problems or effects of donkeys with wounds?
