# Supplementary material for: Evaluating the Efficacy of Knowledge-Transfer Interventions on Animal Health Knowledge of Rural Working Equid Owners in Central Ethiopia: A Cluster-Randomized Controlled Trial
Source: Front Vet Sci. 2018 Nov 20;5:282. doi: 10.3389/fvets.2018.00282 (PMC6256087; doi:10.3389/fvets.2018.00282)
Supplement: Supplementary file 2 [file Data_Sheet_2.PDF]

Working Equids Education Project

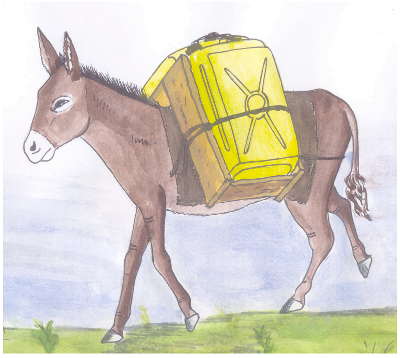

LONG TERM FOLLOW UP QUESTIONNAIRE

ID Number:

Name:

Date:

Gender:

DA Name:

Village (Kebele):

Method of education:

C	HO	VM	AO

Section 1: Wounds:

1. On which body part of the donkey that you are observing manmade wounds most frequently? (*Picture aid*)

2. What are the causes of manmade wounds of donkeys?

3. Tell us two signs observed on donkeys before a wound is occurring?

Section 2: Wound treatment:

4. What care should be taken before you wash the wounds of donkeys?

5. Tell us the best way that you should use to cure the wounds of donkey?

6. Tell us the bad ways that should not be used to treat the wounds of a donkey?

7. To wash wound of donkeys, how much salt and how much water should be mixed?

8. How many times a day you should wash wound of donkeys?

Section 3: Prevention:

9. What type of “baselayer” you have to use for your donkeys?

10. To make the harness good and protect the donkey from the load tell us 3 things you should observe?

11. For the “baselayer” of the donkeys what care should be taken?

12. What are the problems or effects of donkeys with wounds?

Section 4: Evaluation:

13. What is the main message you have learnt from this education?

14. Was there anything in the education that you would do now for your donkey?

15. Was there anything in the education that you would not do now, and why would you not do it now?

16. Have you changed your donkey's baselayer since our education?

17. What did you think about the handout/audio programme/education presentation that we gave you about wounds on donkeys?

18. What was easy to understand/clear about the "education"? (ALL FORMATS)

19. What did you not understand/not clear about the "education"? (ALL FORMATS)

20. Have you passed the information in the education on to anyone else? Who?

21. Did you read the handout? If no, did anyone read the handout to you? Who? Do you still have the handout? (HANDOUT VILLAGES ONLY).

22. Any other information about your donkey that you would like to tell us?
