# Supplementary material for: Evaluating the Efficacy of Knowledge-Transfer Interventions on Animal Health Knowledge of Rural Working Equid Owners in Central Ethiopia: A Cluster-Randomized Controlled Trial
Source: Front Vet Sci. 2018 Nov 20;5:282. doi: 10.3389/fvets.2018.00282 (PMC6256087; doi:10.3389/fvets.2018.00282)
Supplement: Supplementary file 3 [file Data_Sheet_3.PDF]

Supplementary Information 3

Table 3: Descriptive statistics of binary responses to individual questions at pre and (138-196 days) post intervention across different interventions.

Responses at long term		Incorrect at pre and post-intervention n (%)	Correct at pre and post-intervention n (%)	Correct at pre-intervention and incorrect at post-intervention n (%)	Incorrect at pre-intervention and correct at post-intervention n (%)
**Question 1**	**C**	114 (91.2)	2 (1.6)	2 (1.6)	7 (5.6)
	**A**	64 (58.7)	3 (2.8)	0 (0.0)	42 (38.5)
	**VM**	26 (20.3)	3 (2.3)	0 (0.0)	99 (77.3)
	**HO**	20 (17.5)	4 (3.5)	0 (0.0)	90 (78.9)
	**All**	224 (47.1)	12 (2.5)	2 (0.4)	238 (50.0)
**Question 2**	**C**	124 (99.2)	1 (0.8)	0 (0.0)	0 (0.0)
	**A**	106 (97.2)	0 (0.0)	0 (0.0)	3 (2.8)
	**VM**	111 (86.7)	0 (0.0)	1 (0.8)	16 (12.5)
	**HO**	96 (84.2)	0 (0.0)	0 (0.0)	18 (15.8)
	**All**	437 (91.8)	1 (0.2)	1 (0.2)	37 (7.8)
**Question 3**	**C**	125 (100.0)	0 (0.0)	0 (0.0)	0 (0.0)
	**A**	109 (100.0)	0 (0.0)	0 (0.0)	0 (0.0)
	**VM**	127 (99.2)	0 (0.0)	0 (0.0)	1 (0.8)
	**HO**	110 (96.5)	0 (0.0)	0 (0.0)	4 (3.5)
	**All**	471 (98.9)	0 (0.0)	0 (0.0)	5 (1.1)
**Question 4**	**C**	122 (97.6)	0 (0.0)	1 (0.8)	2 (1.6)
	**A**	87 (79.8)	2 (1.8)	0 (0.0)	20 (18.3)
	**VM**	60 (46.9)	0 (0.0)	0 (0.0)	68 (53.1)
	**HO**	60 (52.6)	2 (1.8)	0 (0.0)	52 (45.6)
	**All**	329 (69.1)	4 (0.8)	1 (0.2)	142 (29.8)
**Question 5**	**C**	104 (83.2)	1 (0.8)	1 (0.8)	19 (15.2)
	**A**	22 (20.2)	6 (5.5)	0 (0.0)	81 (74.3)
	**VM**	11 (8.6)	0 (0.0)	0 (0.0)	117 (91.4)
	**HO**	14 (12.3)	3 (2.6)	0 (0.0)	97 (85.1)
	**All**	151 (31.7)	10 (2.1)	1 (0.2)	314 (66.0)
**Question 6**	**C**	124 (99.2)	0 (0.0)	1 (0.8)	0 (0.0)
	**A**	100 (91.7)	0 (0.0)	1 (0.9)	8 (7.3)
	**VM**	49 (38.3)	0 (0.0)	0 (0.0)	79 (61.7)
	**HO**	26 (22.8)	1 (0.9)	0 (0.0)	87 (76.3)
	**All**	299 (62.8)	1 (0.2)	2 (0.4)	174 (36.6)

Key: C = Control, A = Audio, VM = Village Meeting, HO = Handout, All = All interventions

Table 3 (continued): Descriptive statistics of binary responses to individual questions at pre and (138-196 days) post intervention across different interventions.

Responses at long term		Incorrect at pre and post-intervention n (%)	Correct at pre and post-intervention n (%)	Correct at pre-intervention and incorrect at post-intervention n (%)	Incorrect at pre-intervention and correct at post-intervention n (%)
**Question 7**	**C**	123 (98.4)	0 (0.0)	2 (1.6)	0 (0.0)
	**A**	100 (91.7)	1 (0.9)	0 (0.0)	8 (7.3)
	**VM**	76 (59.4)	1 (0.8)	0 (0.0)	51 (39.8)
	**HO**	44 (38.6)	0 (0.0)	1 (0.9)	69 (60.5)
	**All**	343 (72.1)	2 (0.4)	3 (0.6)	128 (26.9)
**Question 8**	**C**	88 (70.4)	10 (8.0)	19 (15.2)	8 (6.2)
	**A**	59 (54.1)	8 (7.3)	20 (18.3)	22 (20.2)
	**VM**	60 (46.9)	11 (8.6)	20 (15.6)	37 (28.9)
	**HO**	52 (45.6)	7 (6.1)	10 (8.8)	45 (39.5)
	**All**	259 (54.4)	36 (7.6)	69 (14.5)	112 (23.5)
**Question 9**	**C**	125 (100.0)	0 (0.0)	0 (0.0)	0 (0.0)
	**A**	101 (92.7)	0 (0.0)	8 (7.3)	0 (0.0)
	**VM**	117 (91.4)	0 (0.0)	0 (0.0)	11 (8.6)
	**HO**	59 (51.8)	0 (0.0)	0 (0.0)	55 (48.2)
	**All**	402 (84.5)	0 (0.0)	8 (1.7)	66(13.9)
**Question 10**	**C**	125 (100.0)	0 (0.0)	0 (0.0)	0 (0.0)
	**A**	106 (97.2)	0 (0.0)	2 (1.8)	1 (0.9)
	**VM**	115 (89.8)	1 (0.8)	1 (0.8)	11 (8.6)
	**HO**	106 (93.0)	0 (0.0)	2 (1.8)	6 (5.3)
	**All**	452 (95.0)	1 (0.2)	5 (1.1)	18 (3.8)
**Question 11**	**C**	85 (68.0)	13 (10.4)	14 (11.2)	13 (10.4)
	**A**	48 (44.0)	28 (25.7)	14 (12.8)	19 (17.4)
	**VM**	43 (33.6)	31 (24.2)	11 (8.6)	43 (33.6)
	**HO**	36 (31.6)	23 (20.2)	5 (4.4)	50 (43.9)
	**All**	212 (44.5)	95 (20.0)	44 (9.2)	125 (26.3)
**Question 12**	**C**	125 (100.0)	0 (0.0)	0 (0.0)	0 (0.0)
	**A**	108 (99.1)	0 (0.0)	1 (0.9)	0 (0.0)
	**VM**	125 (97.7(	0 (0.0)	0 (0.0)	2 (2.3)
	**HO**	103 (90.4)	0 (0.0)	0 (0.0)	11 (9.6)
	**All**	461 (96.8)	0 (0.0)	1 (0.2)	14 (2.9)

Key: C = Control, A = Audio, VM = Village Meeting, HO = Handout, All = All interventions
