# Supplementary material for: Evaluating the Efficacy of Knowledge-Transfer Interventions on Animal Health Knowledge of Rural Working Equid Owners in Central Ethiopia: A Cluster-Randomized Controlled Trial
Source: Front Vet Sci. 2018 Nov 20;5:282. doi: 10.3389/fvets.2018.00282 (PMC6256087; doi:10.3389/fvets.2018.00282)
Supplement: Supplementary file 4 [file Data_Sheet_4.PDF]

Supplementary Information 4: Baseline information and comparison across intervention groups for categorical data for 516 participants in a c –RCT in Oromia region, Ethiopia.

			intervention				
**Variable**		**Overall**	**Control**	**Audio**	**Handout**	**Village Meeting**	**Chi square P value**
		(%)	n (%)	n (%)	n (%)	n (%)	
**Education level**	No Education	24.6	26 (20)	30 (25)	34 (27)	37 (27)	0.6
	Adult Education only	14.5	14 (11)	21 (18)	19 (15)	21 (15)	
	Primary	33.3	49 (38)	33 (28)	40 (31)	50 (36)	
	Junior	13.8	19 (15)	18 (15)	15 (12)	19 (14)	
	Higher	13.6	22 (17)	17 (14)	19 (15)	12 (9)	
	Other (Advanced)	0.2	0 (0)	0 (0)	1 (1)	0 (0)	
**Literacy (Oromo)**	No	78.5	101 (78)	99 (83)	102 (80)	103 (74)	0.4
	Yes	21.5	29 (22)	20 (17)	26 (20)	36 (26)	
**Literacy (Amharic)**	No	44.6	50 (39)	53 (45)	56 (44)	71 (51)	0.2
	Yes	55.4	80 (62)	66 (56)	72 (56)	68 (49)	
**Listen to radio daily**	No	20.0	25 (19.2)	23 (19.3)	19 (14.8)	36 (25.9)	0.2
	Yes	80.0	105 (80.8)	96 (80.7)	109 (85.2)	104 (74.1)	
**Number of donkeys**	0	6.0	6 (4.6)	8 (6.7)	7 (5.5)	10 (7.2)	0.04
	1	52.1	60 (46.2)	79 (66.4)	60 (46.9)	70 (50.4)	
	2	27.9	43 (33.1)	22 (18.5)	41 (32.0)	38 (27.3)	
	3	10.3	19 (14.6)	6 (5.0)	13 (10.2)	15 (10.8)	
	>3	3.7	2 (1.5)	4 (3.4)	7 (5.5)	6 (4.3)	
**Own horse**	No	71.1	71 (54.6)	76 (63.9)	109 (85.2)	111 (79.9)	<0.001
	Yes	28.9	59 (45.4)	43 (36.1)	19 (14.8)	28 (20.1)	
**Own mule**	No	97.5	124 (95.4)	116 (97.5)	128 (100)	135 (97.1)	0.1
	Yes	2.5	6 (4.6)	3 (2.5)	0 (0)	4 (2.9)	
**Own cattle/ox**	No	6.4	5 (3.8)	6 (5.0)	11 (8.6)	11 (7.9)	0.3
	Yes	93.6	125 (96.2)	113 (95.0)	117 (91.4)	128 (92.1)	
**Own sheep**	No	37.0	33 (25.4)	37 (31.1)	69 (53.9)	52 (37.4)	<0.001
	Yes	63.0	97 (74.6)	82 (68.9)	59 (46.1)	87 (62.2)	
**Own goat**	No	74.2	107 (83.3)	96 (80.7)	82 (64.1)	98 (70.5)	<0.001
	Yes	25.8	23 (17.7)	23 (19.3)	46 (35.9)	41 (29.5)	
**Own dog**	No	27.9	18 (13.8)	35 (29.4)	36 (28.1)	55 (39.6)	<0.001
	Yes	72.1	112 (86.2)	84 (70.6)	92 (71.9)	84 (60.4)	
**Own Poultry**	No	21.9	30 (23.1)	25 (21.0)	22 (17.2)	36 (25.9)	0.4
	Yes	78.1	100 (76.9)	94 (79.0)	106 (82.8)	103 (74.1)	
